# Synthesis of Si/C Composites by Silicon Waste Recycling and Carbon Coating for High-Capacity Lithium-Ion Storage

**DOI:** 10.3390/nano13142142

**Published:** 2023-07-24

**Authors:** Jinning Huang, Jun Li, Lanxin Ye, Min Wu, Hongxia Liu, Yingxue Cui, Jiabiao Lian, Chuan Wang

**Affiliations:** 1 Institute of Advanced Synthesis, School of Chemistry and Molecular Engineering, Jiangsu National Synergetic Innovation Center for Advanced Materials, Nanjing Tech University, Nanjing 211816, China; hjn18856617552@163.com (J.H.); yeah_0509@163.com (L.Y.); 202061105024@njtech.edu.cn (M.W.); 2 Institute for Energy Research, Jiangsu University, Zhenjiang 212013, China; li42485115@163.com (J.L.); yxcui@ujs.edu.cn (Y.C.); jblian@ujs.edu.cn (J.L.); 3 College of Electrical Engineering and Control Science, Nanjing Tech University, Nanjing 211816, China; lhx_cec@126.com

**Keywords:** waste recycling, silicon scraps, carbon coating, Si/C composites, anode materials

## Abstract

It is of great significance to recycle the silicon (Si) kerf slurry waste from the photovoltaic (PV) industry. Si holds great promise as the anode material for Li-ion batteries (LIBs) due to its high theoretical capacity. However, the large volume expansion of Si during the electrochemical processes always leads to electrode collapse and a rapid decline in electrochemical performance. Herein, an effective carbon coating strategy is utilized to construct a precise Si@C_PPy_ composite using cutting-waste silicon and polypyrrole (PPy). By optimizing the mass ratio of Si and carbon, the Si@C_PPy_ composite can exhibit a high specific capacity and superior rate capability (1436 mAh g^−1^ at 0.1 A g^−1^ and 607 mAh g^−1^ at 1.0 A g^−1^). Moreover, the Si@C_PPy_ composite also shows better cycling stability than the pristine prescreen silicon (PS-Si), as the carbon coating can effectively alleviate the volume expansion of Si during the lithiation/delithiation process. This work showcases a high-value utilization of PV silicon scraps, which helps to reduce resource waste and develop green energy storage.

## 1. Introduction

With the development of the global economy, non-renewable resources such as oil and natural gas can no longer meet people’s needs. Therefore, the development of a series of renewable resources has gradually become a research hotspot, and among them, solar energy has attracted much attention [1]. In recent years, the photovoltaic industry, as the core of solar cells, has mainly developed silicon-based solar cells, meaning that the large-scale photovoltaic (PV) industry inevitably produced abundant silicon (Si) kerf slurry waste, causing the waste of resources and environmental pollution issues [2,3]. Therefore, it is important to find a method that can not only recover the silicon powder in the waste residue of the PV industry, but also to discover anode materials for lithium-ion batteries with excellent electrochemical performance.

LIBs are widely used as energy storage devices in daily life, and their advantages are having a high energy density and a long cycle life. With the increasing energy density of LIBs, the supply of traditional carbon-based materials has fallen short in the energy market. Therefore, the development of rationally designed new anode materials has become a core research topic [4,5,6,7,8]. Much research has focused on the development of high-capacity active materials and components to improve the energy density and performance of next-generation LIBs. Among them, silicon has a high theoretical specific capacity, which is ten times that of graphite. Silicon-based materials, which are more prominent among non-carbon-based materials, have become the most promising candidates for the new generation of lithium-ion battery anodes due to their high specific capacity [9,10], abundant natural reserves, and environmental friendliness [11]. Conversely, despite the advantages mentioned above, in the process of lithiation/delithiation, the volume of silicon inflate will reach 120–400% [4], resulting in the crushing of silicon particles, the splitting and dispersion of electrode materials, and the damage of electrode structure, thus affecting the long-term cycle stability of lithium-ion batteries. Meanwhile, the volume effect will cause the rupture of silicon and the continuous formation of new solid electrolyte interphase (SEI) layers [12], resulting in a decline of Coulombic efficiency (CE), an increase in battery internal resistance, poor rate performance, poor conductivity, and battery failure, thus limiting the commercial application of silicon anodes. Therefore, it is still a huge challenge to design silicon composites [13].

For the practical application of power batteries to meet the requirements of higher energy density and fast charging of batteries, it is required that the design of Si/C materials should fully consider the specific capacity per unit volume and the structural stability of the material under high current impact [14,15]. At the same time, attention should be paid to improving the cycle life and poor conductivity of silicon anode materials. Although it is an effective way to slow down the volume expansion by adjusting the pore structure, it will also lead to low tap density and thus low battery volume capacity [16]. Therefore, carbon coating becomes an effective method to alleviate the stress caused by volume expansion and increase the conductivity of silicon materials [17,18,19,20,21,22,23,24,25,26]. In addition, the first step towards commercialization is to reduce costs so that they can be used in production. Consequently, it is essential to seek simple, inexpensive, and pollution-free silicon raw materials and to develop simple and effective preparation processes.

Crystalline silicon cutting waste silicon raw-Si from Shangrao Jinko Photovoltaic Power Generation Co, Ltd, Jiangxi Province, China (particle size is 1–100 µm) was ball-milled, pickled, and calcined to remove the organic compounds in the waste silicon powder, and the structure remained unchanged, small particle flakes of prescreen silicon (PS-Si) with micron/irregular superimposition were presented. The long-cycle performance and stability of raw-Si and PS-Si as LIBs anode materials at 0.1 A g^−1^ for 300 cycles were investigated. The charge–discharge specific capacities of the waste silicon before treatment were 356 mAh g^−1^ and 1073 mAh g^−1^, respectively. After pretreatments such as pickling and ball milling, the charge–discharge specific capacities were 1397 mAh g^−1^ and 3106 mAh g^−1^, respectively, and the ICE was also increased from 33% to 44%. The content of impurities such as metal elements and organics was high in the waste silicon before being treated, which reduced the capacity of silicon (theoretical capacity is 4200 mAh g^−1^), and after 20 cycles, the specific capacity of both dropped to about 50 mAh g^−1^. However, after pretreatment, the first charge–discharge specific capacity and ICE were improved. The main reason for the low capacity is due to the significant volume expansion in the process of silicon lithiation; during the continuous cycle of lithiation/delithiation in LIBs, silicon repeatedly expands and contracts, and the electrodes are pulverized, which eventually leads to the attenuation of the lithium storage capacity. This work not only increases the value of waste silicon and reduces the resource waste, but also promotes the development of high-capacity anode materials for green energy storage.

## 2. Experimental

### 2.1. Synthesis of Silicon Hydroxylation

As shown in Figure S1, 15 mL of H_2_O_2_ was slowly added to 45 mL of concentrated H_2_SO_4_, and then placed in an oil bath and heated to 80 °C. Afterward, prescreen silicon (PS-Si) suspension was hydroxylated on its silicon surface, then washed with water and dried to obtain PS-Si-OH with hydroxyl groups attached to the surface.

### 2.2. Synthesis of Mesoporous Silicon/Carbon Composites

An appropriate amount of PS-Si-OH powder was dissolved in deionized water, and then 200 µL, 400 µL, and 800 µL of polypyrrole (PPy) were added into the above solution, respectively. After cooling for 30 min, 100 mg, 200 mg, and 400 mg of ammonium persulfate were poured into the above solution, respectively. After stirring in the ice bath for 12 h, the products were obtained and named Si@PPy-1, Si@Ppy-2, Si@Ppy-3, respectively. The Si@Ppy were centrifugally washed with water and alcohol, and then dried. Finally, Si@Ppy were heated to 400 °C for 1 h under an N_2_ atmosphere. The Si@C_Ppy_-1, Si@C_Ppy_-2, and Si@C_Ppy_-3 composites were obtained.

Detailed descriptions about characterization methods and electrochemical measurements are given in the supplementary material.

## 3. Results and Discussion

**Figure 1:**
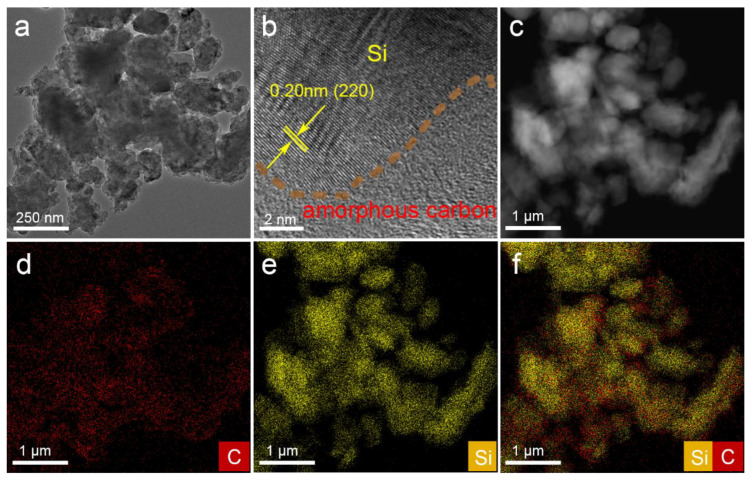
(**a**) TEM, (**b**) HRTEM, (**c**) HAADF, and (**d–f**) elemental mapping images of Si@C_Ppy_-2.

The morphology of Si@C_Ppy_ composites were examined by scanning electron microscopy (SEM). As shown in Figure S2a–c, Si@C_Ppy_-1, Si@C_Ppy_-2, and Si@C_Ppy_-3 present nanosheet-like structures. Compared with the SEM image of PS-Si (Figure S3), the carbon material is coated on the surface of silicon nanosheets and maintains a sheet-like structure. To better understand the structure of Si@C_Ppy_-2 composites, high-resolution TEM and energy dispersive X-ray spectroscopy (EDX) tests were carried out. As shown in Figure 1b, the Si nanoflakes are coated with an amorphous carbon layer from the carbonization of Ppy, which reduces the volume expansion of Si. In addition, as shown in the EDX element mapping (Figure 1c–f), the uniform distribution of Si and C is consistent with the uniform carbon layer coated on the surface of Si nanosheets.

**Figure 2:**
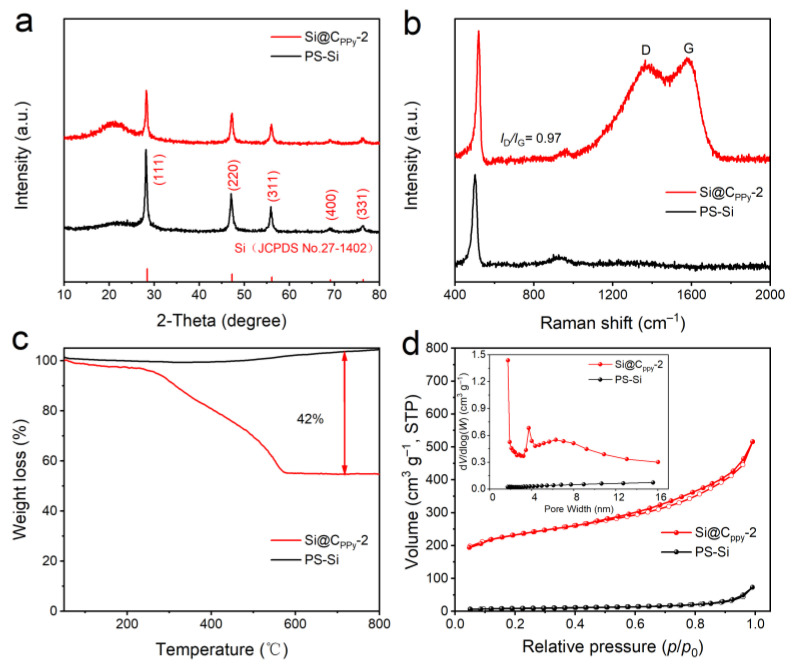
(**a**) XRD patterns, (**b**) Raman spectra, (**c**) TGA curves, and (**d**) N_2_ adsorption–desorption isotherms with the pore size distribution of PS-Si and Si@C_Ppy_-2.

To prove that the carbon was successfully coated on the silicon surface, XRD and Raman tests were performed. Figure 2a and Figure S4a show five peaks at 2θ = 28°, 47°, 56°, 69°, and 76°, corresponding to (111), (220), (311), (400), and (331) of Si, respectively [27]. Additionally, the peak at 32° is assigned to SiO_2_ caused by surface oxidation. The D and G peaks are characteristic Raman peaks at 1300 cm^−1^ and 1580 cm^−1^; among them, the D peak represents the defect degree of carbon itself, and the G peak represents the degree of graphitization and the stretching vibration of the carbon material. The larger *I*_D_/*I*_G_ value shows the lower degree of graphitization [28,29,30,31,32]. As displayed in Figure 2b and Figure S4b, the *I*_D_/*I*_G_ values of Si@C_Ppy_-1, Si@C_Ppy_-2, and Si@C_Ppy_-3 composite are 1.03, 0.97, and 1.06, respectively, showing that the Si@C_Ppy_-2 material has a higher degree of graphitization and the existence of amorphous carbon [33], which can provide more reactive sites for Li^+^ and enhance electronic conduction, and thus improve the ability to store Li^+^. To estimate the carbon content in the composites, thermogravimetric analysis (TGA, Figure 2c) was performed in air. The weight of the composites starts to decrease at 300 °C and decreases by 42 wt.% at 600 °C, corresponding to the carbon content of Si@C_Ppy_-2. A large amount of Ppy carbon material is successfully coated on the surface of silicon waste by simple chemical oxidative polymerization. To optimize the carbon content, Si@C_Ppy_-1 and Si@CPPy-3 composites were synthesized. In comparison to the Si@C_Ppy_-2 composite, Si@C_Ppy_-1 and Si@C_Ppy_-3 composites exhibit similar morphologies (Figure S2a,c), but have different carbon weight ratios (10.12 wt.% for Si@C_Ppy_-1 and 32.2 wt.% for Si@C_Ppy_-3, Figure S5). After carbon coating of PS-Si, the type of N_2_ adsorption/desorption curve for Si@C_Ppy_-2 is type IV, indicating that there are a large number of mesoporous structures (Figure 2d and Table S1). Mesopores can alleviate the volume change of Si by strain relaxation to ensure the structural integrity, thus improving the cycle stability [34].

**Figure 3:**
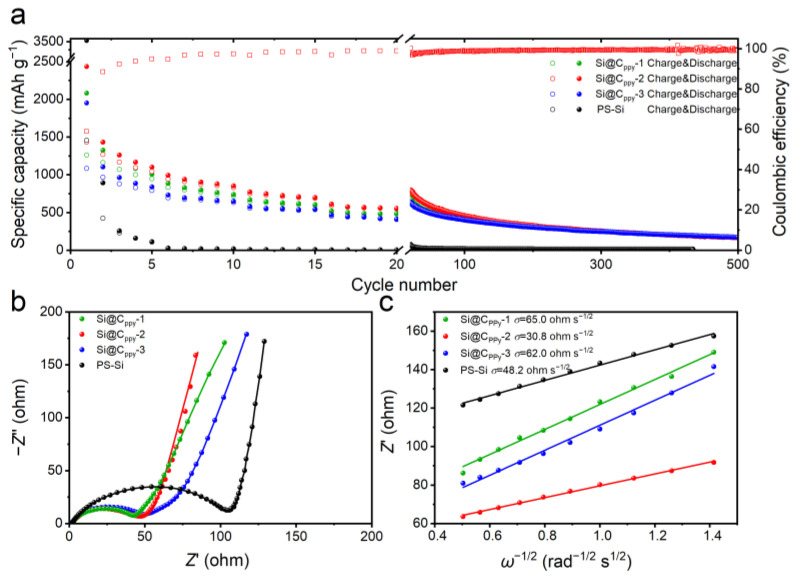
Electrochemical performance of Si@C_PPy_ electrodes in lithium-ion half-cells. (**a**) Rate and cycling performance. (**b**) Nyquist plots. (**c**) Fitting *Z′* and *ω*^−1/2^.

To verify the electrochemical performance of composites, lithium-ion half-cells were assembled with Si@C_Ppy_-1, Si@C_Ppy_-2, and Si@C_Ppy_-3 anode materials, respectively. The charge–discharge tests of Si@C_Ppy_ and PS-Si anode materials were carried out at the same current density. As shown in Figure 3a, the Si@C_Ppy_-2 composite can maintain a high specific capacity of 335 mAh g^−1^ and a coulombic efficiency of 99% after 200 cycles. Furthermore, the Si@C_Ppy_-2 composite has a high specific capacity of 1436 mAh g^−1^ at 0.1 A g^−1^, and a high capacity of 607 mAh g^−1^ even at 1.0 A g^−1^. At a higher current density, the discharge capacity of Si@C_Ppy_ is higher than that of PS-Si, indicating a better rate performance of Si@C_Ppy_. In addition, when the current density is restored to 0.1 A g^−1^, the specific capacity of Si@C_Ppy_-2 can still reach 778 mAh g^−1^, showing its excellent structural stability. Compared with silicon anode materials, the carbon-coated silicon composites achieve higher reversible capacity and CE values, which can be attributed to the improved mechanical stress and the stability of SEI layers. 

To further study the electrical resistance and lithium diffusion kinetics of Si@C_Ppy_-2 composites, EIS tests were performed. The equivalent circuit and Nyquist plots are shown in Figure 3b. The Nyquist diagrams include a semicircle and an oblique line in the high-frequency and low-frequency regions, respectively, which correspond to the charge transfer resistance (*R*_ct_) and the Warburg impedance, respectively [35,36]. As shown in Table S2, the *R*_ct_ value of Si@C_Ppy_-2 (38.85 ohm) is relatively low, indicating the strong charge transfer ability of Si@C_Ppy_-2. In addition, by fitting *Z′* to *ω*^−1/2^ in a low-frequency *Z*_w_ region (Figure 3c), the Warburg factor (*σ*) for PS-Si, Si@C_Ppy_-1, Si@C_Ppy_-2, and Si@C_Ppy_-3 is 48.2 ohm s^−1/2^, 65.0 ohm s^−1/2^, 30.8 ohm s^−1/2^, and 62.0 ohm s^−1/2^, respectively. The ion diffusion coefficient is further calculated by *σ*, and its calculation formula is as follows:

(1) $$D_{{Li}^{+}}=\;\frac{R^{2}T^{2}}{2A^{2}n^{4}F^{4}C^{2}\sigma^{2}}$$

where *σ*, *ω*, *R*, *T*, *A*, *n*, *F*, and *C* stand for the Warburg factor, angular frequency, gas constant, absolute temperature, electrode surface area, transfer electron number per molecule, Faraday constant, and Li^+^ molar concentration, respectively. The *D*_Li_^+^ of Si@C_PPy_-2 electrode material is about 39.56 times that of Si@C_PPy_-1 and 4.45 times that of Si@C_PPy_-3, respectively, showing that the Li^+^ diffusion time of the Si@C_PPy_-2 electrode is shorter than that of the Si@C_PPy_-1 and Si@C_PPy_-3 composites. The electron transfer speed and ion diffusion kinetics of Si@C_PPy_-2 are faster than other electrodes, which indicates that the addition of a suitable amount of carbon can improve the rate performance of Si-based electrodes.

**Figure 4:**
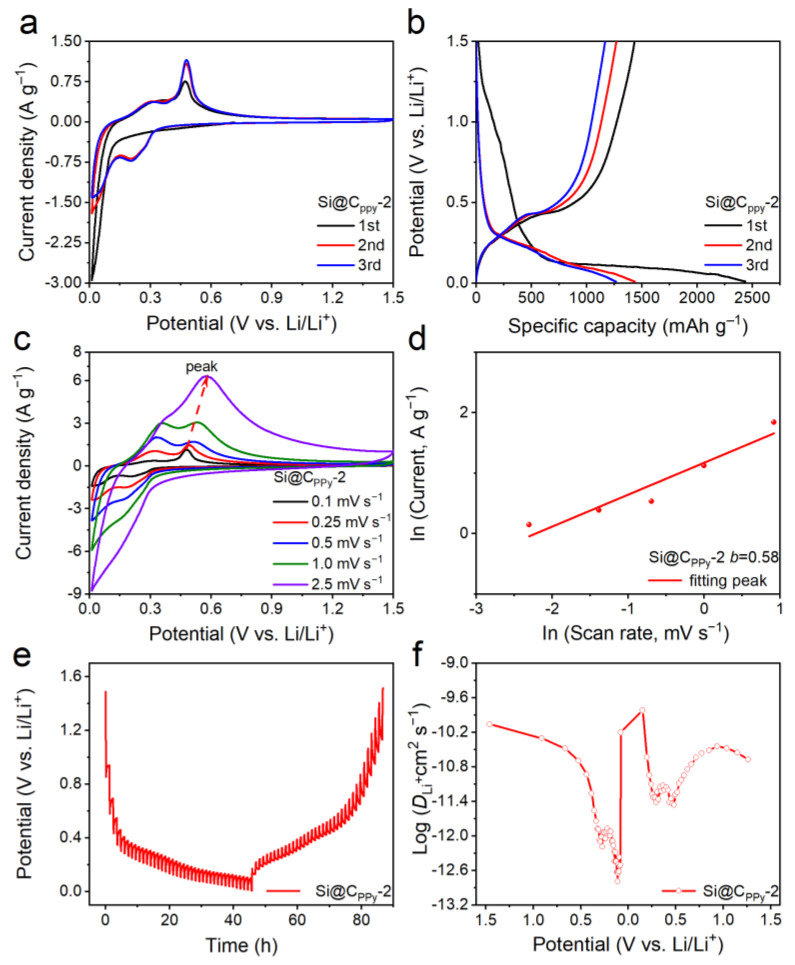
Electrochemical performance of Si@C_PPy_-2 electrode in lithium-ion half-cells. (**a**) CV curves at a scan rate of 0.1 mV s^−1^. (**b**) The initial three GCD curves at a current density of 0.1 A g^−1^. (**c**) CV curves at various scan rates from 0.1 to 2.5 mV s^−1^. (**d**) *b*-value determination. (**e**) GITT curve. (**f**) Lithium-ion diffusion coefficient.

The electrochemical performance of Si@C_PPy_-2 was explored using cyclic voltammetry (CV) curves. Figure 4a shows the first three CV curves of Si@C_PPy_-2 composites with a scan rate of 0.1 mV s^−1^ and a voltage window of 0.01−1.5 V. There is a wide reduction peak at 0.2V in the first cathodic scan due to the formation of SEI layer [31]. Moreover, there may be some side reactions between the electrolyte and electrode materials, leading to the increase in irreversible charging capacity in the first cycle. In the subsequent cycles, the reduction peak at 0.2 V represents the alloying process of crystalline silicon to lithium silicon, while the two oxidation alloying peaks near 0.3 V and 0.5 V represent the de-alloying process of the Li*_x_*Si phase as follows [34,35,36]:

Si + *x*Li^+^ + *x*e^−^ ⇋ Li*_x_*Si (2)

Figure 4b and Figure S6 exhibit the galvanostatic discharge–charge (GCD) profiles of Si@C_PPy_ composites for the initial three cycles measured at a current density of 0.1 A g^−1^ between 0.01 and 1.50 V (vs. Li/Li^+^). The first discharge specific capacity is 2083, 2433, and 1950 mAh g^−1^ for Si@C_PPy_-1, Si@C_PPy_-2, and Si@C_PPy_-3, respectively. Meanwhile, the first charge capacities of 1259, 1431, and 1083 mAh g^−1^ were obtained for Si@C_PPy_-1, Si@C_PPy_-2, and Si@C_PPy_-3, respectively. Si@C_PPy_-1, Si@C_PPy_-2, and Si@C_PPy_-3 composites achieve an ICE of 60.4%, 58.8%, and 55.5%, respectively. It is known that the theoretical capacity of amorphous carbon is around 600 mAh g^−1^, suggesting that the addition of a moderate amount of slightly graphitized carbon could suppress the fracture of silicon by increasing the electronic conductivity and deactivation of silicon anode materials. Hence, the Si@C_PPy_-2 composite delivers a higher specific capacity.

To deeply study the storage kinetics of lithium ions, the CV curves of the three composites at different scan rates were recorded. As shown in Figure 4c and Figure S7, the CV curves of the three samples were similar, with one reduction peak and two oxidation peaks (from 0.1 to 2.5 mV s^−1^). The peak current (*i*) and the scan rate (*v*) have a relationship according to Equation (3), where *i* is the peak current, *v* is the scan rate, *a* and *b* are constants. The *b* value can be calculated from the slope of log(*i*)–log(*v*) and reflect the electrochemical reaction kinetics of Li-ion batteries.

*i* = *av*^*b*^ (3)

log(*i*) = *b*log(*v*) + log(*a*) (4)

It is worth pointing out that *b* = 0.5 indicates diffusion-controlled reactions, while *b* = 1 means surface-controlled reactions. As shown in Figure 4d, the *b* value of the Si@C_PPy_-2 composite is 0.58, which is closer to 0.5, suggesting that the diffusion-dominating process controls the lithium storage dynamics.

The galvanostatic intermittent titration technique (GITT) was applied to investigate the diffusion coefficient of Li-ions (*D*_Li_^+^) by applying a pulse current at 0.1 A g^−1^ for 10 min and standing for 1 h (Figure 4e). The *D*_Li_^+^ can be calculated by the formula as follows:

(5) $$D_{{Li}^{+}}=\frac{4}{\pi \tau }{\left(\frac{m_{b}V_{M}}{M_{B}S}\right)}^{2}{\left(\frac{{∆E}_{S}}{∆E_{\tau }}\right)}^{2}$$

where *m*_b_ and *M*_B_ represent the active mass and molar mass, respectively. *τ* represents the pulse time, and *V_M_* and S represent the molar volume of the electrode sheet and the surface area of the active mass, respectively. Additionally, ∆*E_S_* and ∆*E_τ_* represent pulse-induced voltage changes and galvanostatic charge–discharge voltage changes, respectively [37]. As shown in Figure 4f, in the lithiation process, the *D*_Li_^+^ of the Si@C_PPy_-2 composite is 10^−9.5^−10^−13^ cm^2^ s^−1^. These values are 100 times that of PS-Si. It can be seen that the Si@C_PPy_-2 composite has faster diffusion kinetics [38,39]. In short, the good electrochemical performance of the Si@C_PPy_-2 composite is attributed to its unique structural features: (i) porous structure and protective carbon layer can help to slow down the volume changes of silicon during lithiation/delithiation, and (ii) the large specific surface area (SSA) can provide sufficient electrode/electrolyte contact area, while the porous structure facilitates electrolyte penetration and ion transport, thereby accelerating Li^+^ diffusion transfer during the discharge-charge process. Therefore, carbon coating is beneficial for improving the electrochemical performance of PS-Si and ensuring the integrity of the overall structure of the anode electrode material, thereby achieving an excellent lithium storage capacity.

## 4. Conclusions

In summary, Si@C_PPy_ composites are successfully fabricated by annealing PPy. The carbon layer can alleviate the pulverization and expansion problem of Si during the lithiation/delithiation process, leading to a stable electrochemical performance. In addition, the carbon coating can increase the electrode/electrolyte contact area by increasing the SSA of the anode material. Moreover, the Si@C_PPy_ composites possess mesoporous structures and excellent electrical conductivity, which can deliver an excellent cycling performance. The Si@C_PPy_-2 composite can still exhibit a high specific capacity of 460 mAh g^−1^ after 100 cycles, while the specific capacity of PS-Si is only 18 mAh g^−1^. Hence, this work not only provides an effective way for the high-value utilization of PV Si waste, but also opens a new strategy for designing alloying-type anode materials for energy storage.

## Data Availability

Not applicable.

## Supplementary Materials

The following supporting information can be downloaded at: https://www.mdpi.com/article/10.3390/nano13142142/s1. Figure S1: Illustration of the preparation process of Si@C_PPy_ materials; Figure S2: SEM of (a) Si@C_PPy_-1, (b) Si@C_PPy_-2, and (c) Si@C_PPy_-3; Figure S3: SEM images of (a) Raw-Si and (b) PS-Si; Figure S4: (a) XRD patterns and (b) Raman spectra of Si@C_PPy_-1 and Si@C_PPy_-3; Figure S5: TGA curves of PS-Si, Si@C_PPy_-1, and Si@C_PPy_-3; Figure S6: (a,b) Discharge–charge profiles of Si@C_PPy_-1 and Si@C_PPy_-3 at 0.1 A g^−1^; Figure S7: (a,b) CV curves of Si@C_PPy_-1 and Si@C_PPy_-3 at various scan rates; Table S1: BET specific surface area, pore volume, and BJH pore size of PS-Si and Si@C_PPy_-2; and Table S2: *R*_s_ and *R*_ct_ values of Si@C_PPy_-1, Si@C_PPy_-2, and Si@C_PPy_-3.
