# Supplementary material for: Synthesis of Si/C Composites by Silicon Waste Recycling and Carbon Coating for High-Capacity Lithium-Ion Storage
Source: Nanomaterials (Basel). 2023 Jul 24;13(14):2142. doi: 10.3390/nano13142142 (PMC10386753; doi:10.3390/nano13142142)
Supplement: Supplementary file 1 [file nanomaterials-13-02142-s001.zip › nanomaterials-2469054-supplementary.pdf]

Supplementary Material

For

Synthesis of Si/C composites by silicon waste recycling and carbon coating for high-capacity lithium-ion storage

Jinning Huang [1,†], Jun Li [2,†], Lanxin Ye [1], Min Wu [1], Hongxia Liu [3], Yingxue Cui [2], Jiabiao Lian [2] and Chuan Wang [1,*]

1 Institute of Advanced Synthesis, School of Chemistry and Molecular Engineering, Jiangsu National Synergetic Innovation Center for Advanced Materials, Nanjing Tech University, Nanjing 211816, China; hjn18856617552@163.com (J.H.); yeah_0509@163.com (L.Y.); 202061105024@njtech.edu.cn (M.W.)

2 Institute for Energy Research, Jiangsu University, Zhenjiang 212013, China; li42485115@163.com (J.L.); yxcui@ujs.edu.cn (Y.C.); jblian@ujs.edu.cn (J.L.)

3 College of Electrical Engineering and Control Science, Nanjing Tech University, Nanjing 211816, China; lhx_cec@126.com

* Correspondence: ias_cwang@njtech.edu.cn

† These authors contributed equally to this work.

Experimental Section

Characterizations Methods: The morphologies of the samples were observed by scanning electron microscopy (SEM, JEOL JSM-7800) and transmission electron microscopy (TEM, JEOL JEM-2100F). The crystalline structures of the products were examined using X-ray diffraction (XRD, German Bruker D8) with Cu Kα radiation. Raman spectra were characterized on a Raman system (JY Company HR800) with a 532 nm laser. The carbon content in the composites was determined employing a thermogravimetric analysis (TGA, Netzsch TSA 449 F3). The N_2 adsorption-desorption isotherms were tested by a gas absorption analyzer (Micrometric TriStar Ⅱ3020). The specific surface area and pore size distribution was calculated by the Brunauer-Emmett-Teller (BET) method and Barrett-Joyner-Halenda (BJH) model, respectively.

Electrochemical Measurements: The working electrodes were prepared by mixing the active materials, Ketjen black (KB), and polyvinylidene fluoride (PVDF) binder at a weight ratio of 8:1:1 in N-methylpyrrolidone (NMP). After stirring for 1 h, The homogeneous slurries were obtained and pasted onto the copper foil current collectors, and then dried at 60 °C under vacuum for 12 h. After that, the above copper foils were roll-pressed at a pressure of 10 MPa and punched into disks with a diameter of 12 mm. The mass loading of the active materials was about 1.0 mg cm^{-2}. The CR2032 coin-type cells were assembled in an argon-filled glovebox using Li foils, polypropylene membranes (Celgard 2400), and 1.0 M $LiPF_6$ in ethylene carbonate (EC)/dimethyl carbonate (DMC) (1:1 in volume ratio) as the counter electrodes, separators, and electrolyte, respectively. The galvanostatic charging/discharging (GCD) and cyclic voltammetry (CV) tests were conducted on the Neware battery testing system (CT-4008) and Gamry electrochemical workstation (IN-TERFACE500P) in a voltage window of 0.01–1.50 V (*vs.* Li/Li^+), respectively. The electrochemical impedance spectroscopy (EIS) was performed using a Gamry electrochemical workstation (INTERFACE500P) with the frequency range from 100 kHz to 10 mHz at an open circuit potential with an AC perturbation of 10 mV. The galvanostatic intermittent titration technique (GITT) measurements were tested using a Neware battery testing system (CT-4008) at a current density of 0.1 A g^{-1} and a pulse time of 10 min between 1 h rest intervals.

Supplementary Figures:

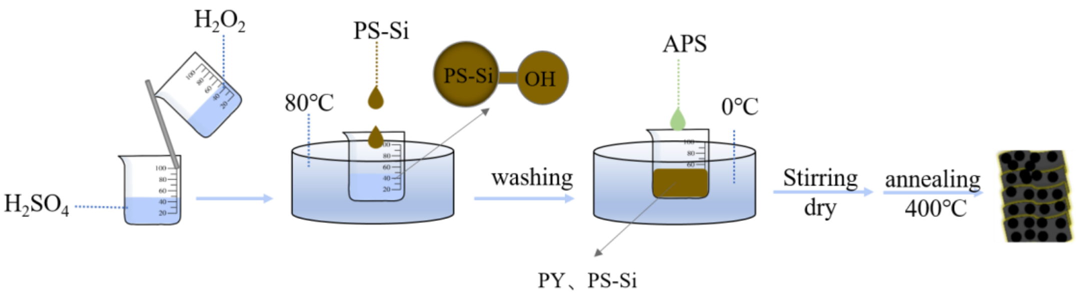

Figure S1. Illustration of the preparation process of Si@C$_{PPy}$ materials.

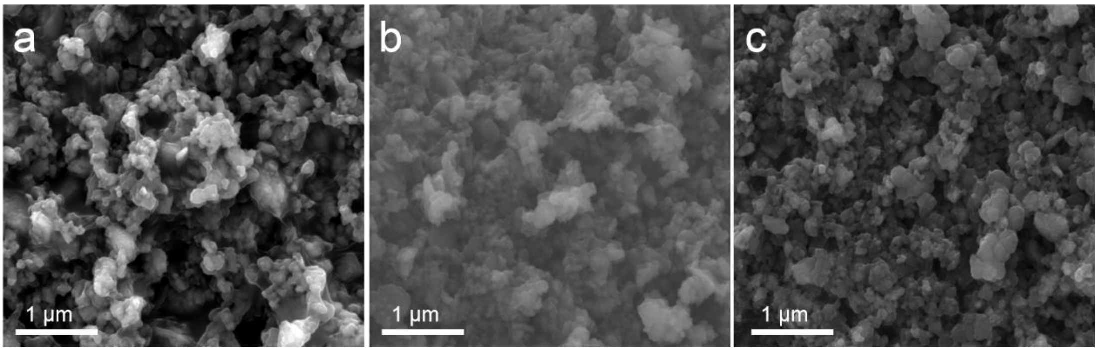

Figure S2. SEM of (**a**) Si@C_{PPy}-1, (**b**) Si@C_{PPy}-2, and (**c**) Si@C_{PPy}-3.

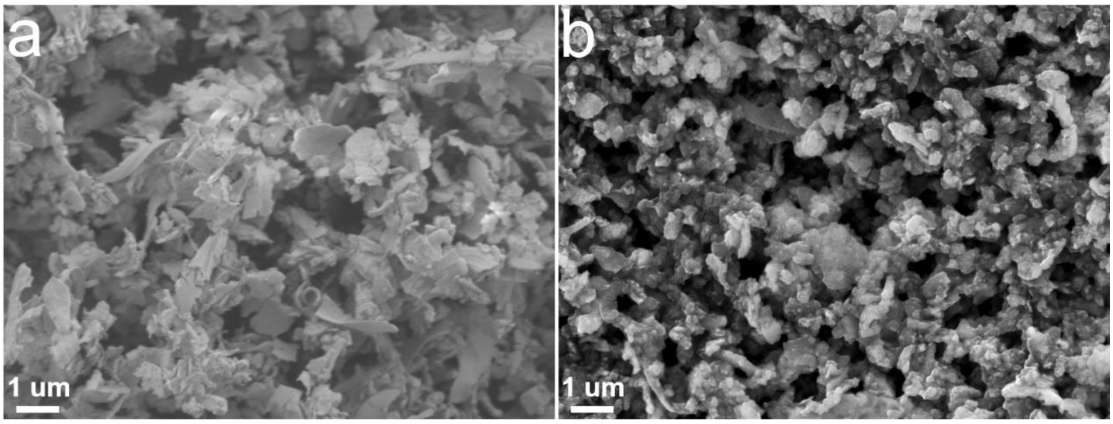

Figure S3. SEM images of (**a**) Raw-Si and (**b**) PS-Si.

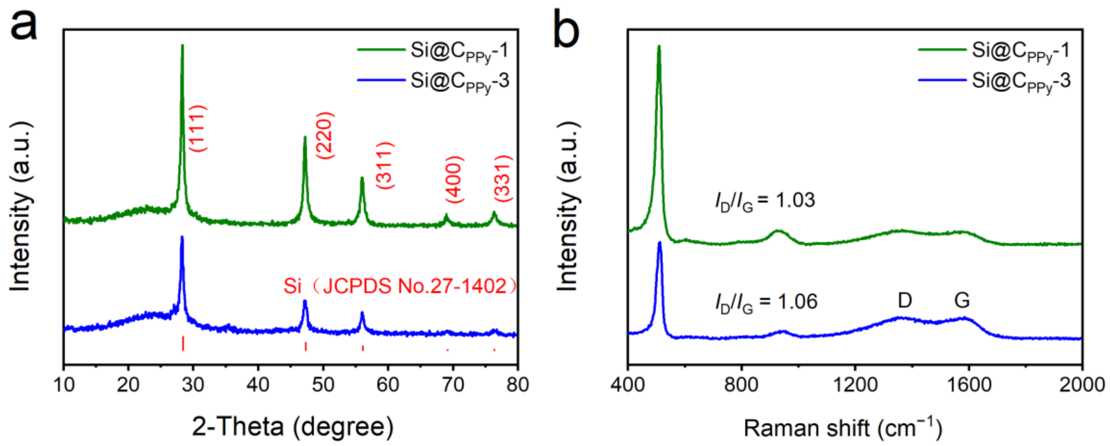

Figure S4. (**a**) XRD patterns and (**b**) Raman spectra of Si@C$_{PPy}$-1 and Si@C$_{PPy}$-3.

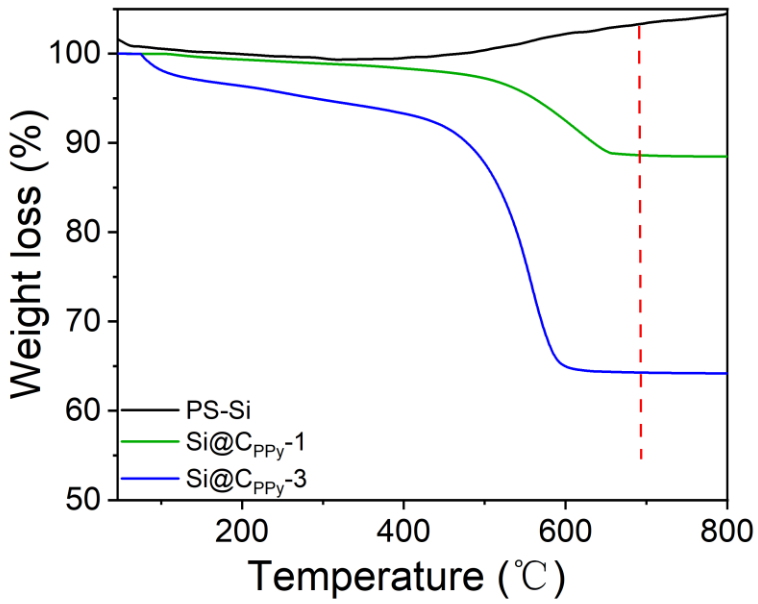

Figure S5. TGA curves of PS-Si, Si@C_{PPy}-1 and Si@C_{PPy}-3.

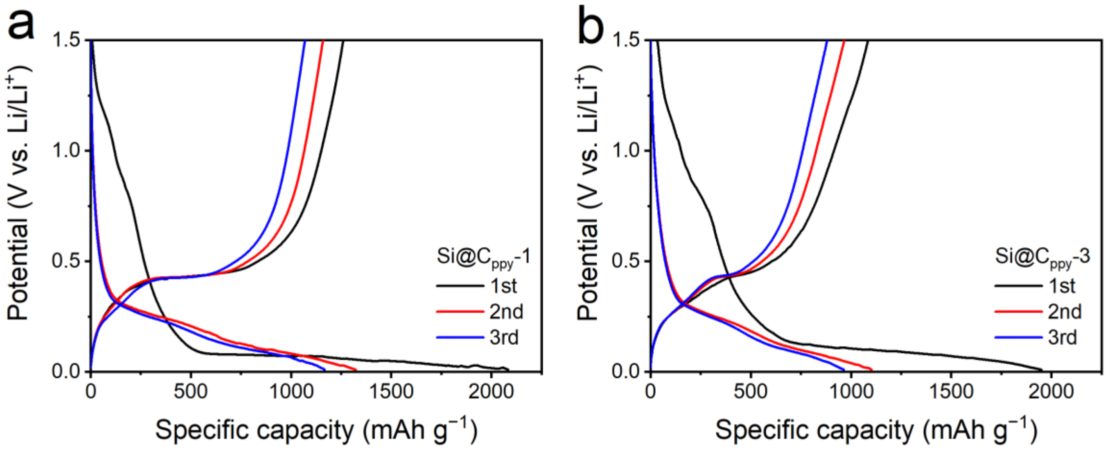

Figure S6. (**a, b**) Discharge-charge profiles of Si@C$_{PPy}$-1 and Si@C$_{PPy}$-3 at 0.1 A g^{-1}.

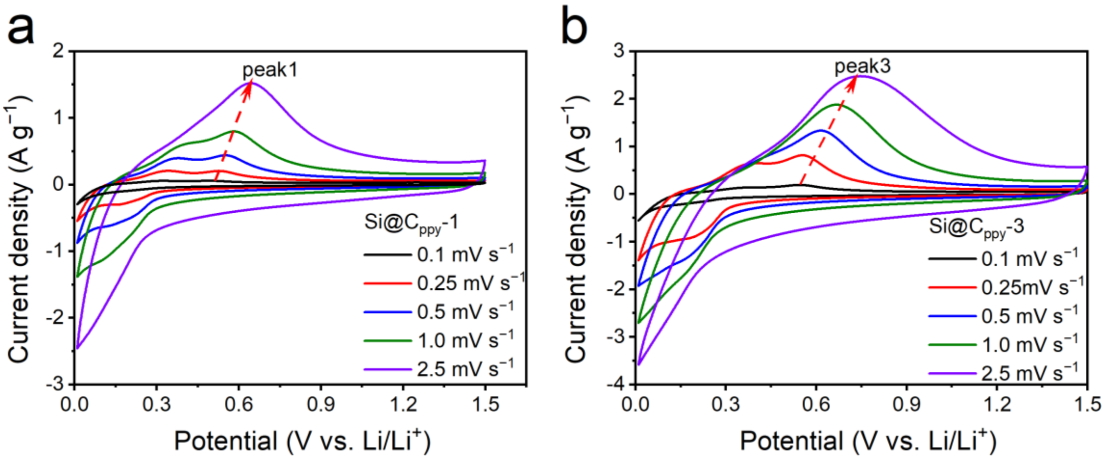

Figure S7. (**a, b**) CV curves of Si@C$_{PPy}$-1 and Si@C$_{PPy}$-3 at various scan rates.

Supplementary Tables:

Table S1. BET specific surface area, pore volume, and BJH pore size of PS-Si and Si@C_{PPy}-2.

Sample	S_{BET} (m^2 g^{-1})	V_{total} (cm^3 g^{-1})	Average pore size（BJH） (nm)
PS-Si	31.3	0.044	5.83
Si@C_{PPy}-2	753	0.504	3.84

Table S2. The R_s and R_{ct} values of Si@C_{PPy}-1, Si@C_{PPy}-2, and Si@C_{PPy}-3.

Samples	R_s (ohm)	R_{ct} (ohm)
Si@C_{PPy}-1	1.53	34.48
Si@C_{PPy}-2	1.65	38.85
Si@C_{PPy}-3	1.38	40.64
